# Autopolicy: Automated Traffic Policing for Improved IoT Network Security

**DOI:** 10.3390/s20154265

**Published:** 2020-07-30

**Authors:** Pawel Foremski, Sławomir Nowak, Piotr Fröhlich, José Luis Hernández-Ramos, Gianmarco Baldini

**Affiliations:** 1 Institute of Theoretical and Applied Informatics of the Polish Academy of Sciences (IITiS PAN), Gliwice 44100, Poland; snowak@iitis.pl (S.N.); pfrohlich@iitis.pl (P.F.); 2 European Commission, Joint Research Centre (JRC), 21027 Ispra, Italy; jose-luis.hernandez-ramos@ec.europa.eu (J.L.H.-R.); gianmarco.baldini@ec.europa.eu (G.B.)

**Keywords:** Internet of Things, security, sensor networks, traffic policing, Distributed Denial of Service, packet filtering, firewall, Software-Defined Networking

## Abstract

A 2.3Tbps DDoS attack was recently mitigated by Amazon, which is a new record after the 2018 GitHub attack, or the famous 2016 Dyn DNS attack launched from hundreds of thousands of hijacked Internet of Things (IoT) devices. These attacks may disrupt the lives of billions of people worldwide, as we increasingly rely on the Internet. In this paper, we tackle the problem that hijacked IoT devices are often the origin of these attacks. With the goal of protecting the Internet and local networks, we propose Autopolicy: a system that automatically limits the IP traffic bandwidth—and other network resources—available to IoT devices in a particular network. We make use of the fact that devices, such as sensors, cameras, and smart home appliances, rarely need their high-speed network interfaces for normal operation. We present a simple yet flexible architecture for Autopolicy, specifying its functional blocks, message sequences, and general operation in a Software Defined Network. We present the experimental validation results, and release a prototype open source implementation.

## 1. Introduction

Large Distributed Denial of Service (DDoS) attacks that are caused by multitude of hijacked Internet of Things (IoT) devices are currently one of the most important threats to the Internet [1]. Botnets comprised of hundreds of thousands of popular end-devices, like video cameras or home routers, became an everyday reality to administrators of world’s largest and, thus, most important, Internet services—for instance, the DNS [2]. Poor software quality, no automated update procedures, and the use of default authentication credentials make it easy to remotely exploit IoT devices.

Some of these simple techniques for hijacking Internet hosts were identified decades ago, yet they remain profitable until now, with no effective remedy deployed Internet-wide. Moreover, the trend of increasingly pervasive IoT technology is continuing, with billions of devices projected for installation in the coming years [3]. Will the Internet survive new bursts of gigantic DDoS attacks—and more importantly—how? Should we, as the Internet community, keep waiting until our devices are finally secure, or should we rather distrust all IoT devices by default, restricting their Internet access the moment they are plugged into the network?

In this paper, we tackle the challenge that many IoT devices are equipped with powerful CPUs and high-speed network interfaces, which, in general, is desirable, but it has a risky side effect of enabling these devices to send IP packets to arbitrary Internet hosts at very large traffic rates. For instance, the IEEE 802.11ax (Wi-Fi 6) standard will allow for transmission speeds well exceeding 1 Gbit/sec [4]. In this context, we argue that the wide-spread use of a default, unconstrained network access policy makes botnet-launched, volumetric DDoS attacks feasible. IoT devices rarely need the full network bandwidth or require access to the whole Internet IP addressing space.

Thus, we propose Autopolicy: a system that can dramatically reduce some potentially disastrous effects that hijacked IoT devices can have on the Internet and on their local networks. Autopolicy requires each device in a network to obey a strict set of rules on its generated IP traffic, for instance, the maximum consumed bandwidth and the set of contacted IP addresses. It resembles the concept of a firewall, but in contrary it is largely automatic, primarily applies to upstream traffic, and it takes advantage of the intrinsic features of IoT-generated traffic. It also resembles the concept of anomaly detection, but, on the contrary, it is much simpler, does not rely on statistical models, and prevents attacks instead of detecting them. When deployed, Autopolicy can reduce the size of potential DDoS attacks that originated in a network by several orders of magnitude.

Autopolicy was designed for the needs of the SerIoT project [5], as one of its edge security components, securing the network from potentially malicious endpoints. SerIoT aims at managing information security of IoT platforms and networks in a holistic, cross-layer manner—integrating IoT sensors, devices, honeypots, Software-Defined Networking (SDN) routers, and controllers. The resulting platform can be used to implement secure IoT ecosystems and networks.

The major contributions of this paper are as follows:

- We propose an automated system that can systematically limit the bandwidth (and other network resources) available to IoT devices in a particular network. Our primary goal is to protect networks from IoT devices (from their potentially malicious upstream traffic).
- The system comprises of authentication, authorization, and traffic policy layers, each of which is highly extensible. We present a practical architecture, which allows for automatically building traffic profiles, in case an IoT device does not directly support our proposal.
- We describe a simple solution to prevent potential privilege escalation attacks on Autopolicy, which relies on MAC addresses alone, and does not require additional mechanisms, such as MACsec or WPA (for wired and wireless networks, respectively).
- We provide an open source, prototype implementation of the proposed system, which is designed for seamless operation in a Software-Defined Network (SDN) under the control of the Open vSwitch (OVS) software. The software is implemented in Golang for easy portability to ARM and MIPS-based hardware platforms (see https://github.com/iitis/autopolicy/).
- We present the initial experimental results of deploying Autopolicy in a network testbed, which demonstrates its efficacy in preventing DDoS attacks.

The rest of this paper is organized, as follows: first, we discuss the context of our proposal in Section 2. Subsequently, we give a high-level overview of Autopolicy in Section 3, which can help the reader to understand the following sections that focus on a specific aspect of the system: device identification discussed in Section 4, traffic profiles discussed in Section 5, and policy enforcement in Section 6 (which also presents the prototype implementation and experimental results). We discuss our results in Section 7, and conclude with a short summary in Section 8.

## 2. Background

IoT devices are designed with the aim of of providing more and more functionality, but security is not adequately considered, thus many popular IoT devices are badly protected. It is possible for an attacker to create a botnet by infecting vulnerable devices with malware. Many different types of IoT malware were used in the past in order to exploit security weaknesses and build large-scale botnets, whih are composed of millions of victim hosts: Mirai, Wirex, Reaper, Torii, 3ve-2018 [6]. From a security perspective, the IoT revolution presents a potential disaster. Attacked devices and botnets are often used for cyber-attacks—foremost, the DDoS attacks. A DDoS attack is based on the fact that its victim has limited network resources, which can be saturated by a number of coordinated users. The attack succeeds when the total resources of a botnet exceed those of the victim.

Mirai botnet [7] was created using malware that infected a huge number of IoT devices. Mirai infection started by a rapid scanning phase, where asynchronous TCP-SYN probes were sent to pseudo-random IPv4 addresses, on Telnet TCP ports 23 and 2323. Potential victims were attacked using brute-force login attemps, trying to establish a Telnet session while using a predefined list of popular usernames and passwords. Successful logins were reported to a hardcoded server. The vulnerable devices, after logging in, were infected by downloading and executing architecture-specific malware, turning the device into a bot listening for attack commands. The botnet size has changed over time, reaching 600,000 devices at its peak and 300,000 devices on average. It was used to perform over 15,000 DDoS attacks, and the botnet was even offered for rent by the criminals behind it.

As an example of attacks that were performed using Mirai, let us consider the Dyn cyberattack [2]. It was a series of DDoS attacks, carried out on October 21 2016, targeting DNS systems. The attack caused major Internet platforms and services to become unavailable to users in Europe and North America. The attack was performed through numerous DNS lookup requests from the botnet, which consisted of many Internet-connected devices such as printers, IP cameras, residential gateways and baby monitors, all infected with the Mirai malware.

Although Mirai was not the only botnet used to perform attacks, it serves as a call to secure IoT devices and to develop mechanisms and standards on the security and privacy of an IoT-enabled world. Potential future Mirai-like botnets can be used for ad fraud, camera extortion, bitcoin mining, or any number of applications. IoT devices tend to be ideal targets for potential use as botnets, since there are millions of devices with several security problems:

- they have exposed administrative ports protected by weak passwords;
- owners often do not have the knowledge to secure their devices;
- popular, cheap IoT devices are created by manufacturers which do not provide system updates in terms of security—or, even if the updates are available—they are not carried out by the unaware device owners;
- despite limited connection requirements—e.g., sending periodic measurements—these devices often are equipped with fast network interfaces; and,
- there is no common practice of limiting their bandwidth for security purposes, and no automatic control mechanisms that are dedicated to IoT devices.

The problem of how to mitigate the security risks related to the Internet of Things, including DDoS attacks, is widely discussed in the literature. However, there are no common standards that relate to infrastructure protection against DDoS attacks that are directed from a large number of devices [8]. The recommendations and best practices that have been formulated [9] are also not widely deployed.

In [10], the authors present an analysis of recent research in IoT security, including trends, open issues, and new technologies, e.g., blockchain. Reference [11] gives a comprehensive analysis on the security issues in IoT: the authors state that SDN could contribute significantly to the security, but the paper focuses on managing encryptions keys. The paper [12] reviews IoT security solutions that are based on SDN technology in a detailed way: the authors argue that SDN-based architectures have many advantages, but also point out that the separation of the control and data plane in SDN networks results in low packet processing performance, resulting in packet delay or loss.

Network operators are responsible for security of not only local systems, but also the entire infrastructure. Organizations must focus on controlling and securing their entire IoT systems. It is recommended to develop IoT security systems that enable automated policies [9]. For instance, in [13], the authors point out the necessity to correlate the information from multiple security components, and that IoT security systems should communicate directly, without or with limited human intervention.

The basic approach is to provide access limitation on specific interfaces with some context awareness according to specific environmental conditions [14]. In [15], a number of access control methods in the Internet of Things are presented. A more complex system that is capable of automatically identifying the types of IoT devices and enabling enforcement of rules for constraining the communications is described in [16]. This approach focuses on the device-type identification technique for IoT devices. Devices are assigned into certain isolation classes, however that work is not focused on managing more complex traffic profiles.

Another possible approach is to implement protocol-level access control (instead of network traffic verification). An example for the MQTT pub/sub messaging is shown in [17], following the attribute-based access control (ABAC) model [18]. Although the goals are different (network protection vs. service protection), there are some similarities in the proposed architecture to our paper.

A more relevant approach, where vendors can offer different software and appliances to enforce the network access rules is presented in [19]. The authors propose an automated approach to derive network security policies. However, the work focuses on the design of a standardized network security policy, but does not specify the details on how to solve each aspect, e.g., how to obtain policies for a given device.

In [20], the authors discuss a possible solution to DDoS attacks in SDN-based networks that uses an external Intrusion Detection System (IDS) that is based on Anomaly Detection (AD). The IDS is responsible for analyzing IP flows that have no SDN rules associated. As a result, the SDN controller creates new rules that allow or (in case of an attack) block the flow. Similar to Autopolicy, the mechanism is based on automatic acquisition of traffic profiles. The solution is universal and can be implemented in non-SDN networks to a limited extent, e.g., home networks. However, Autopolicy is not an AD system: instead of analyzing traffic, it enforces clearly defined traffic limits obtained for given device identity. Our solution is effective in preventing attacks and requires less resources (no AD traffic analysis).

Recently, the IETF published RFC 8520 [21]: a standard for Manufacturer Usage Description (MUD). While similar, our work was created independently and aims at protecting the Internet and local networks, whereas MUD (among the others) aims at protecting the devices by providing a means to address their security vulnerabilities faster than through system updates. When comparing with MUD, Autopolicy explicitly targets DDoS attacks by limiting the available IP traffic bandwidth, and it offers more visibility into IoT devices through the concept of device identity files.

## 3. Autopolicy Overview

**Figure 1:**
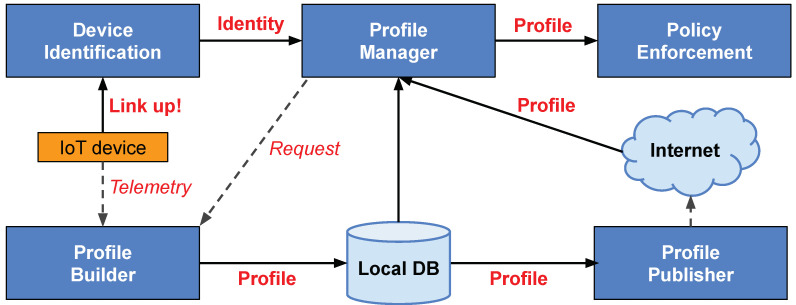
Overview of Autopolicy: function blocks (blue) and information flow (red).

The general design of Autopolicy is presented in Figure 1, which illustrates the functional blocks and basic flow of information in the system. In this picture, the IoT device is trying to connect to the network, bringing its network interface up, which is detected by its SDN switch—e.g., when receiving the first frame from a new MAC address. So far, all IP traffic from and to device is blocked, apart from the protocols necessary for link auto-configuration—e.g., Dynamic Host Configuration Protocol (DHCP) and Link Layer Discovery Protocol (LLDP).

Next, the Device Identification (DI) function of the system authenticates the device, by discovering its “Identity”, i.e., a set of key-value entities that describe the device manufacturer, model name, and other characteristics (see Section 4). The information collected by DI—along with the device MAC address—is then forwarded to the Profile Manager (PM).

The objective of PM is to authorize the device and find its Traffic Profile, i.e., a set of allowed and disallowed characteristics of transmitted IP traffic (see Section 5). In order to find the profile, PM queries a local database (*Local DB* in Figure 1), or fetches the profile from the Internet, e.g., over the HTTPS protocol. If needed, PM can request the Profile Builder to create a tailor-made traffic profile for the device, based on IP traffic statistics collected in an external system, e.g., using IPFIX [22].

Finally, a restricted network access is granted. The profile is sent to the Policy Enforcement (PE) function, which allows the flow of IP traffic under the set of rules specified in the profile. This function is normally realized by the SDN switch that started the whole procedure, i.e., the switch directly connected with the IoT device. PE is the crucial step of Autopolicy, as this is where we actually prevent attacks (see Section 6).

### Protocol Sequences

**Figure 2:**
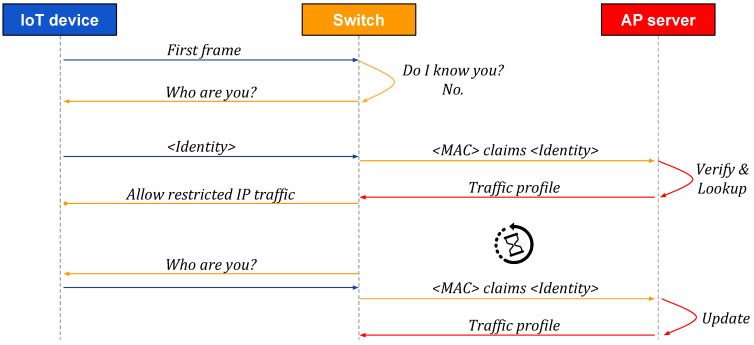
Sequence of messages: an IoT device that supports Autopolicy connects to a protected network. The switch forwards its identity file to the Autopolicy server (AP server), which verifies it and replies with a traffic profile. The procedure is periodically repeated for possible profile updates.

**Figure 3:**
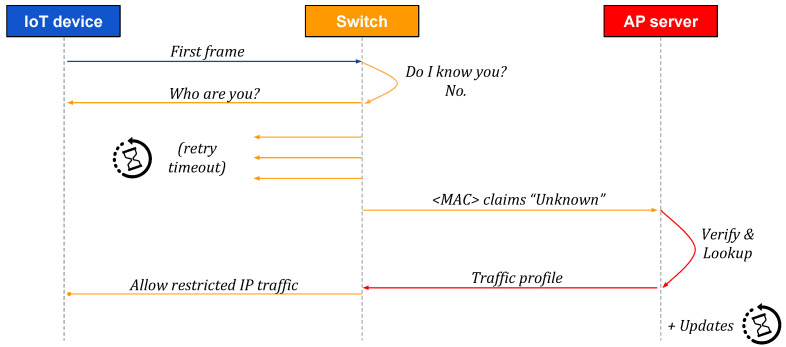
Sequence of messages: a non-conforming IoT device connects to an Autopolicy network. The switch synthesizes an "Unknown" identity, and sends it to the Autopolicy server (AP server), which replies with a generic traffic profile that restricts network resource usage.

**Figure 4:**
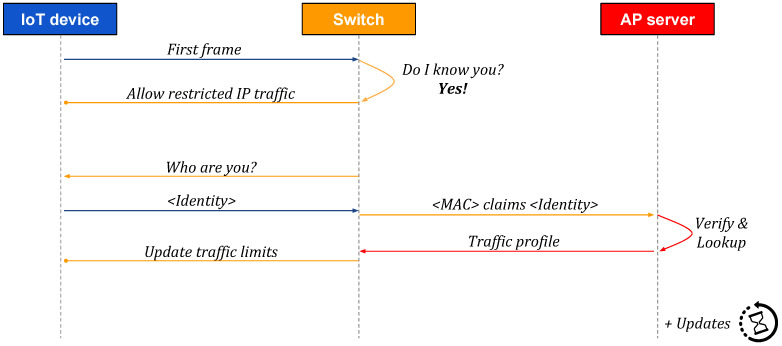
Sequence of messages: the switch has all required information on the connecting IoT device in its local cache of the already seen devices, and thus can immediately admit the device to the network. The standard procedure continues asynchronously, in order to fetch possible updates to the traffic profile.

Figure 2, Figure 3 and Figure 4 present high-level sequences of network messages and actions between an IoT device, its SDN switch, and an Autopolicy server (AP server). Each figure reflects different circumstances and demonstrates how the switch responds to a new IoT device connecting to an Autopolicy network.

First, in Figure 2, the IoT device supports Autopolicy and provides its identity declaration when queried by the switch (see Section 4). The switch forwards the identity and MAC address to the AP server and gets the corresponding traffic profile back (see Section 5), which will immediately enable the flow of IP traffic for the IoT device (see Section 6).

On the other hand, in Figure 3, the IoT device is not aware of Autopolicy, and it does not respond to identity queries made through any of the supported protocols. In such a case, the switch retries the queries a few times before failing, effectively waiting a given amount of time for the device to respond. Because the only piece of information that we have in such a case is the device MAC address, we create an empty “Unknown” identity for the device, and proceed as in the previous case.

Finally, in Figure 4, we consider the situation in which the switch has already seen the same MAC address on the same port, foremost in the case the device reboots due to maintenance (e.g., firmware upgrade). In order to reduce the delay before the IoT device is allowed to transmit IP packets, the switch applies the most recently seen traffic profile for the device, learned previously, as discussed for Figure 2 and Figure 3. Nevertheless, we continue with the standard procedure for authentication and authorization, and update the traffic limits if needed.

## 4. Device Identities

One of the main tasks of Autopolicy is device authentication, i.e., learning the identity of the device and making sure it cannot be spoofed. The basic method we use is to query the device for its identity file, and to trust the information submitted on the very first connection with the network. Further changes to the already learned identity are restricted to only a few parameters (detailed later).

The key information we use to track a device is its MAC address. Note that MAC addresses, in general, can easily be spoofed. As a countermeasure, before a new MAC address can be utilized in the network, we require it to be approved, only for particular switch and one of its ports. Moreover, once we learn the association between a particular MAC address and device identity, the binding must not be removed or replaced without explicit approval.

### 4.1. Identity Files

An identity file is a JSON-encoded file holding an object that describes an IoT device. The purpose of an identity file is to let Autopolicy know where to obtain the traffic profiles from, and to help administrators in managing the network. In Listing 1, we show an illustrative identity file.

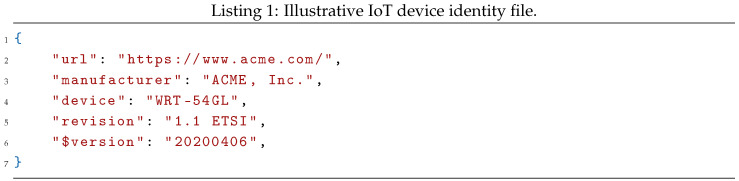



   The possible parameters are as follows (see also Section 4.4):

1 url: the root HTTPS URL under which the device manufacturer publishes traffic profiles for its IoT devices; this is usually the only required parameter, which must always specify an HTTPS URL with a valid PKI certificate,
2 manufacturer: manufacturer name,
3 device: device name, e.g., model name,
4 revision: device revision, e.g., model revision code,
5 $version: identity version, expressed as a UTC timestamp (see Section 4.4).

Identity files are extensible, allowing for implementing new functionality in future. For instance, one could add a parameter for fetching traffic profiles using other than HTTPS means, e.g., using a blockchain-based approach. However, all of the parameters must be JSON strings comprised of the ASCII character set, and all values must be directly mappable to the ASCII character set, in order to facilitate compatibility with alternative mechanisms for learning the device identity.

### 4.2. Identity Queries

An Autopolicy-enabled switch must monitor all of its ports for new source MAC addresses. Whenever the port link state changes, the list of MAC addresses seen on that port should be flushed. Every new source MAC address seen on a particular port must be authenticated when receiving the first IP packet, and periodically re-authenticated during the connection, e.g., every hour (plus additional random delay).

The goal is to ensure that only legitimate devices are connected, and to prevent unauthorized MAC address changes. We support firmware upgrades by monitoring the link state (which detects device reboots) and re-authenticating connected devices (in case there is no need for reboot). Note that, in general, a new firmware release may need an updated traffic profile.

The switch queries the device and waits for a response, optionally repeating the query, in order to learn the identity of a particular MAC address. In case of no reply after specified time, e.g., 10 s total, the switch generates an empty identity for the device and proceeds. In case the switch already has the traffic profile for particular MAC address in cache, it may immediately enforce it, but must proceed anyway.

The basic method we propose for the identity queries is based on the HTTP protocol:

1 The device configures its IP stack. This is independent from Autopolicy, but note the need to support DHCP and similar mechanisms, which should not be blocked by the switch.
2 The switch learns the IP address of the device by waiting for its first IP, ARP, or ICMPv6 packet (NDP neighbor solicitation [23]).
3 The switch sends an HTTP GET request to http://<IP>/.autopolicy/identity.json, where <IP> is the device IP address (IPv4 or IPv6).
4 The device should respond with a 200 OK status code, and provide the identity file encoded as JSON in the response body.
5 Note that, in the case of re-authentication, the switch must re-learn the IP address of the device, which might have changed since the first authentication attempt.

The main advantage of the basic method that is described above is its simplicity and direct use of the JSON identity file, with no need of translation to different data formats. Note that we deliberately avoid the HTTPS protocol and use plain HTTP instead. Otherwise, we would need a valid PKI certificate in each IoT device, which would cause many scalability and maintenance problems, e.g., due to the amount of work needed to keep the certificate up to date.

We acknowledge that our simple protocol does not guarantee data integrity or confidentiality. However, note that an attacker who controls the direct link between the switch and the device can potentially cause much more harm than just revealing the identity file—which should be considered public anyway—or changing its contents via a Man-in-the-Middle attack. Accordingly, we believe that the proposed basic method is reasonable for many scenarios. Moreover, even if the attacker does change the identity file, in Section 4.4, we describe the identity verification step that minimizes the potential harm.

### 4.3. Advanced Query Methods

Besides the simple HTTP query method described above, Autopolicy supports fetching the identity files over a wide variety of protocols based on standardization efforts, which are analyzed in [24]. Many of these proposals are based on the use of the Extensible Authentication Protocol (EAP) [25] that offers a flexible authentication framework, allowing for the use of different authentication mechanisms, or EAP methods. In such a case, the identity file format can be different than JSON, i.e. any format that supports the key-value paradigm.

In particular, EAP-TLS is based on the use of the well-known Transport Layer Security (TLS) [26], through the use of certificate-based mutual authentication. EAP-TLS is widely used, for example, in the scope of ZigBee IP. Moreover, EAP-PSK is based on the use of a Pre-Shared Key (PSK), so it can be specially considered in constrained devices and networks. Other EAP methods have been recently proposed, such as the Nimble out-of-band authentication for EAP (EAP-NOOB) [27], which is specially aimed to provide a bootstrapping solution for IoT devices that have a minimal user interface and no pre-configured authentication credentials.

For the transport of EAP messages, there have been different proposals according to the network layer responsible for it. One of the main options is related to the use of the Extensible Authentication Protocol over LAN (EAPOL) [28], in which EAP messages are transported through data frames. In this direction, a lightweight version, called Slim EAPOL (SEAPOL), is proposed in [29], in which the bootstrapping process is extended for obtaining authorization credentials.

An alternative is represented by the Protocol for Carrying Authentication for Network Access (PANA) [30]. PANA is widely accepted as a potential candidate for IoT security bootstrapping, and it is used by the ZigBee Alliance in conjunction with EAP-TLS as authentication protocols. A more recent approach proposes the use of the Constrained Application Protocol (CoAP) [31] to transport the EAP messages. The resulting approach, called CoAP-EAP, provides a lightweight, efficient, and flexible alternative to be used in IoT scenarios [32,33].

While it is not the focus of Autopolicy, the identity query method should consider existing technologies for identification purposes, when a new device is deployed on a certain network. It should be noted that the MUD specification [21] defines three different ways to get the MUD URL from the device (DHCP, X.509, LLDP), which may be adapted to fetch the identity file.

### 4.4. Verification

**Figure 5:**
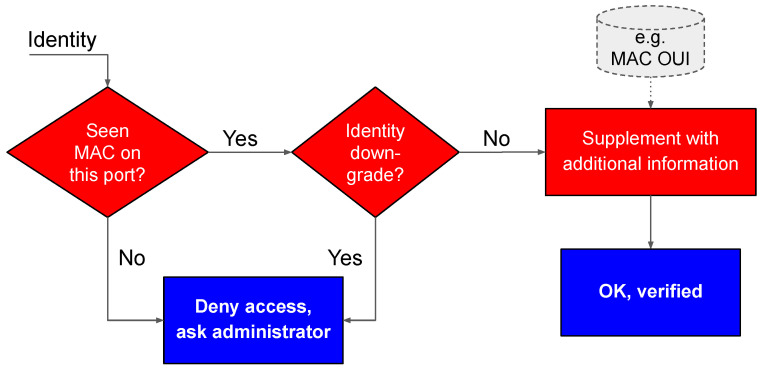
Verifying identity files submitted by IoT devices

The basic sequence of actions for Device Identification was shown in Figure 2 till Figure 4. However, before one can use the identity submitted by an IoT device, it must be verified. Otherwise, an attacker who hijacks the device could forge an identity that would make Autopolicy download arbitrary traffic profiles, thus bypassing all security mechanisms. The basic method that we use for verification is storing the identity we learn when the device connects for the first time, and using it as a reference point. In Figure 5, we illustrate the algorithm.

When the switch obtains the identity, it forwards the JSON object to the Autopolicy server, which must verify the provided data and respond with a traffic profile, or deny network access. The switch must add a few parameters to the forwarded identity:

- @switch: string identifier of the switch, e.g., its hostname;
- @port: name of the switch port the device connects on, e.g., eth1;
- @mac: the MAC address of the device, as seen by switch; and,
- @ip: last seen source IPv4 or IPv6 address for the device (optional parameter).

First, the server checks in a local database if the particular pair of MAC address and port is known, i.e., if the device ever connected to the network at the very same location. If the MAC-port pair is not found in the database, it means we are either dealing with a new device, or the switch port changed, or the MAC address was spoofed (forged by an attacker). In such a case, Autopolicy denies network access, and it notifies network administrator to take action, e.g., to accept the new connection (it can store the submitted identity as a reference point). Optionally, Autopolicy can automatically accept the device if it is the very first connection ever seen on that particular switch port, which, however, should require physical security of the switch ports.

Second, in the case the MAC-port pair is already known, we compare the submitted identity with the one already stored in the local database when the device connected for the first time. We verify whether the submitted identity was not downgraded, using the following procedure:

1 Skip validation for parameters starting with the @ character.
2 Accept all new parameters, i.e., the parameters not present in the already stored identity.
3 For parameters starting with the $ sign, the new value must be greater or the same as the old value when compared lexicographically.
4 Otherwise, the new value must be the same as the old value.

Third, we consult external sources of information, such as the Wireshark manuf file [34], in order to infer more details on the device identity. Note that, if the device did not respond to identity queries and the switch generated an anonymous identity, it should be supplemented in this step. For instance, using the first six bytes of the MAC address one should be able to guess the manufacturer name, which, in turn, may help in fetching the appropriate traffic profile for the device from a local database.

Finally, the verified and supplemented identity file is forwarded to the Profile Manager, described in the next section, which will lookup the traffic profile and send it to the switch.

## 5. Profile Manager

The goal of the Profile Manager (PM) is device authorization, i.e., at this step we already know the device identity, but we still do not know what it is allowed to do. The identities that we receive from the previous step can have a varying level of detail, depending on the device. Thus, the main task of PM is to infer where to download the relevant traffic profile from, which is realized using a hierarchical search algorithm.

We do not put any hard requirements on practical implementation of the search procedure, i.e., traffic profiles can be stored in any abstract database that allows for querying with a flexible level of detail. For instance, this can be implemented while using a directory tree structure, SQL-like RDBMS, public repositories like GitHub, or even blockchain-based distributed systems. We only suggest the system is configurable in such a way that the network administrator can select different sources of traffic profiles for different devices, based on the identity.

In case the system is not able to find the traffic profile for a particular identity, it must be able to provide the requesting switch with a generic, network-wide “default” profile, configured by the network administrator. In case there is no specific profile for a particular device identity, the system can request to create a new traffic profile from the Profile Builder (see Figure 1).

### 5.1. Hierarchical Search

The main idea behind the algorithm is to query the database with the most details we have in an identity, in order to obtain the most specific result. For instance, below we describe the basic, HTTPS-based scheme for fetching traffic profiles:

1 Rewrite all information stored in the identity into the following path:
  PATH = <manufacturer>/<device>/<revision>/<$version>
2 Fetch the traffic profile over HTTPS using the following URL:
  https://<url>/.autopolicy/PATH/profile.json
3 The server must provide a valid SSL certificate (not self-signed) and reply with the HTTP 200 OK response code. In such a case, finish and return the profile.
4 If PATH is empty, finish with error.
5 Otherwise, remove the last section of PATH and repeat from 2.

For example, using the identity that is presented in Listing 1, we would try fetching the profile using the following sequence of URLs (from the most specific to the least specific URL):

1 https://www.acme.com/.autopolicy/acme_inc/wrt-54gl/1_1_etsi/20200406/profile.json
2 https://www.acme.com/.autopolicy/acme_inc/wrt-54gl/1_1_etsi/profile.json
3 https://www.acme.com/.autopolicy/acme_inc/wrt-54gl/profile.json
4 https://www.acme.com/.autopolicy/acme_inc/profile.json
5 https://www.acme.com/.autopolicy/profile.json

If all of the above URLs fail (e.g., due to the HTTP error 404 “Not Found”), we exit the procedure with a general error (no profile fetched). In any case, the Profile Manager should cache the result in order to minimize the number of HTTPS queries sent to the Internet.

Note how in point 1 of the described procedure we convert the values of identity parameters into URI path segments. We convert all letters to lower-case, and allow only for characters commonly found in DNS labels, i.e., latin letters (a–z), digits (0–9), and hyphens (-). All other characters are converted to underscores (_), where groups of adjacent non-allowed characters are converted into a single underscore. Finally, we trim the value left and right, removing all of the prefix and suffix underscores.

The character conversion procedure is needed in order to avoid character encoding ambiguities (e.g., representing the space character either in ASCII or URL-encoded), to minimize the chances of human error (e.g., letter case mismatch), and in general for maximum compatibility with existing systems (by making the path segments resemble filesystem names). Autopolicy implementers should consider the possible URI path collisions, i.e., when two different identity files result in the same URI path (due to the same result of the character conversion procedure). However, in such a case, different device manufacturers will use different url values in the identity file, so the traffic profile will be fetched from different network locations anyway.

### 5.2. Traffic Profiles

Similarly to device identities, we store traffic profiles as JSON files. In Listing 2, we show an illustrative identity file.

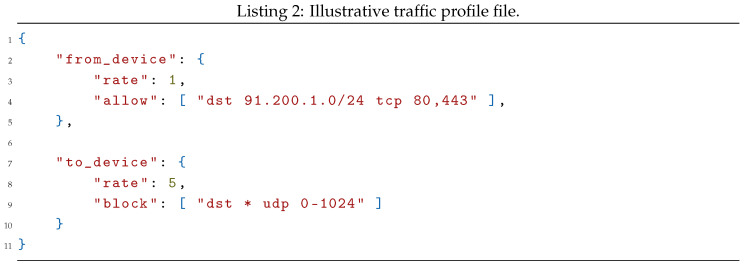



   The traffic profile has 2 JSON objects, named from_device and to_device, which respectively specify traffic policies for IP traffic flowing from the IoT device into the switch port, and flowing from the switch port into the IoT device. For each of these two objects, it is possible to specify the following parameters:

- rate: specifies the maximum bandwidth in Mbit/s (JSON float),
- allow: a list of allowed IP services (JSON array of strings); each value is space-separated:
  1. rule direction, either source (src) or destination (dst);
  2. IP prefix, or * to match any IPv4 or IPv6 address;
  3. transport protocol (tcp or udp), or *;
  4. port number lists and ranges, e.g., 80,443,8000-9000;
  5. as a special case, if there is only one token in the string, it is interpreted as the protocol;
- block: same as the allow above, but for blocking access,
- connections: maximum number of new connection attempts per second (JSON integer),
- src_ips: maximum number of source IP addresses per day (JSON integer); and,
- resolvers: the exclusive set of allowed DNS recursive resolvers (JSON array).

The allow property is exclusive, i.e., all traffic that does not match the rules for a particular direction (from_device or to_device) will be dropped. In case the allow property is not specified at all, and the traffic does not match the block rules, it will be allowed. Autopolicy implementers should carefully prepare their traffic profiles, in order to avoid accidental permission for unwanted IP traffic.

Finally, note that the packet filter is designed to be stateless, i.e., it neither tracks the connections nor automatically opens the firewall for responses to connections initiated by the device.

## 6. Policy Enforcement

When the switch receives the response from its Autopolicy server, it must immediately enforce the traffic policy on the network port, as below:

1 In case the server denied network access, the switch must not allow any IP communication. Instead, it should periodically repeat the same query to the Autopolicy server, in case the administrator accepts the new device in the meantime.
2 In the opposite case, the switch receives a traffic profile, and it must allow IP communication strictly according to the profile contents. It must also periodically repeat the authentication and authorization steps to ensure timely updates of the enforced policy.

For implementing Autopolicy on a Linux SDN switch that is already running the Open vSwitch software [35], we suggest using existing Linux kernel mechanisms, such as the Traffic Control subsystem [36]. Below, we describe a prototype implementation of Autopolicy, which is followed by an experimental validation of the system.

### 6.1. Prototype Implementation

We implemented a proof of concept for our proposal detailed in the previous sections, and released it as open source under https://github.com/iitis/autopolicy.

The project provides two programs: *ap-server*, which implements the Profile Manager function, and *ap-switch*, which implements the Device Identification and the Policy Enforcement functions. For the algorithms and procedures used in the programs, see Figure 1 and Figure 2, Section 4.2 and Section 4.4, and Section 5.1. We chose the Golang language for its performance and easy cross-compilation, in order to target the ARM and MIPS CPU architectures that are used in popular hardware platforms.

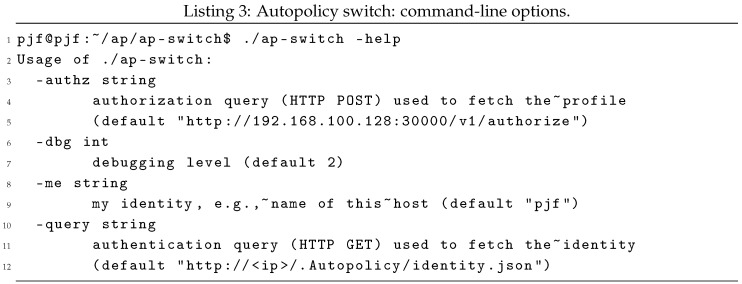



   The ap-switch program requires a list of network interfaces to protect. In Listing 3, we present its command-line options, after which the user provides space-separated list of Linux interface names, e.g., eth0 eth1. When started, ap-switch will first block all IP traffic on controlled ports using tc rules [36], and then start a *sniffer* on these ports, as described in Section 4.2. The sniffer will detect and identify new devices trying to connect, and send queries to the ap-server program running under the URL specified through the -authz command-line option. If a successful response is received, it will install a new set of tc rules implementing the traffic policy for the device. Note that ap-switch uses caching and timeouts to facilitate efficient and reliable operation.

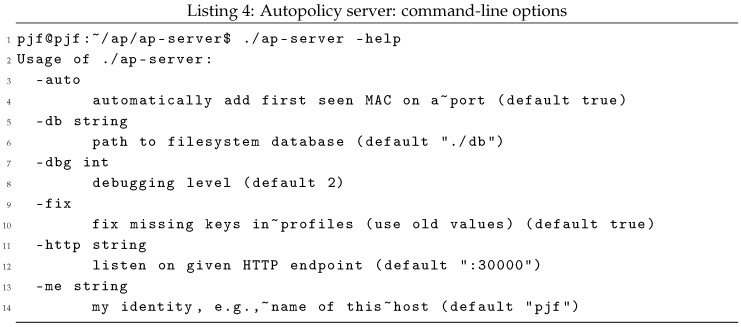



   By default, the ap-server program operates an HTTP server on port 30,000 (configurable parameter, see listing 4) and handles queries sent by ap-switch. It uses a filesystem-based local database, by default under the ./db subdirectory, which follows the following schema:

1 identities/<switch>/<port>/<mac>/: hold reference identities for devices already seen;
2 profiles/<manufacturer>/<device>/<revision>/<$version>/: hold traffic profiles, which are normally downloaded from manufacturer websites and cached for re-use.

The ap-server program can optionally automatically authorize the very first MAC address seen on a new switch port, which reduces the amount of manual work that is needed by the system administrator. This however requires assuming that the first use of a switch port never used before is not malicious, which might not be a reasonable strategy for networks with open access to physical ports. In the usual case, the reference identity will already be stored under ./db/identities, which will allow ap-server to validate the request authenticity and proceed with authorization, as described in Section 5. If a valid traffic profile is already stored under ./db/profiles, it is sent back to the ap-switch program. Otherwise, we use HTTPS to fetch the best matching profile from the manufacturer website.

### 6.2. Autopolicy Testbed

In order to conduct experiments on the Autopolicy prototype, we used a testbed we will describe below: first, as an abstract topology of the network under the test, and second, as a detailed description of the actual solutions used to implement such a topology.

**Figure 6:**
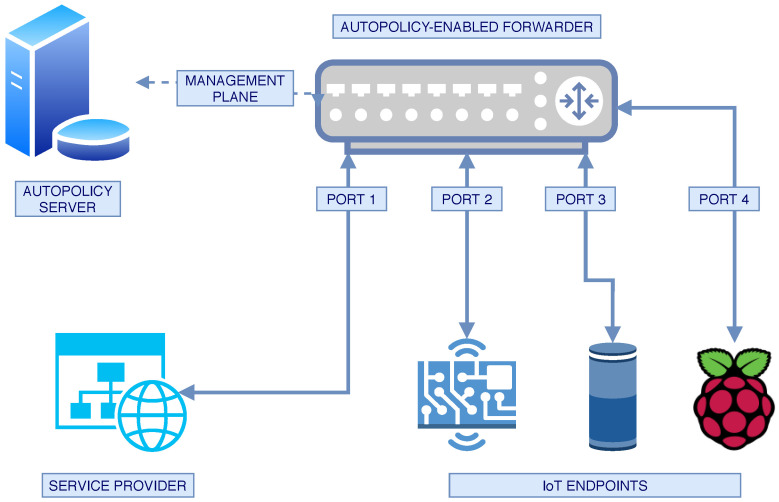
Logical toplogy of the experimental testbed.

**Figure 7:**
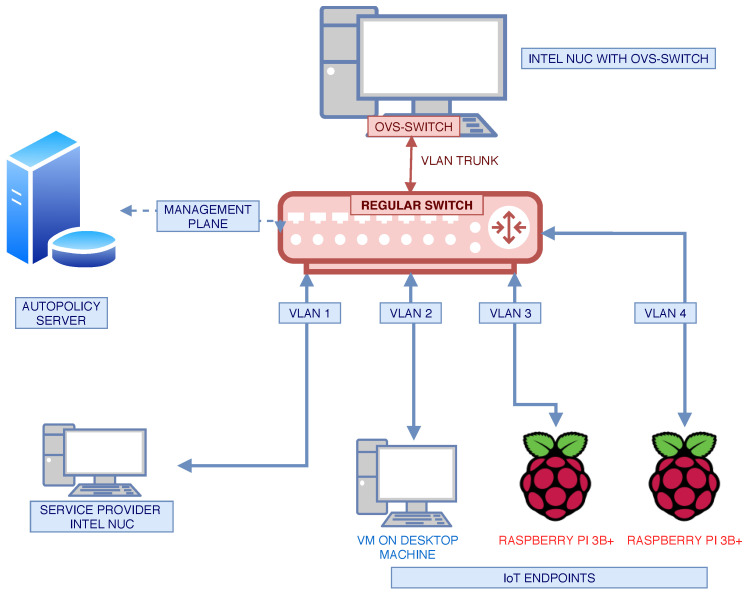
Physical toplogy used to implement the experimental testbed.

The abstract topology is shown in Figure 6 and incorporates an Autopolicy-enabled forwarder (switch) and an Autopolicy server. The service provider and IoT endpoints shown in the figure are not mandatory for Autopolicy to function, but were necessary for the experiments. In Figure 7, we present an implementation of the abstract topology. Note that the placement and naming of the machines correspond to Figure 6. Below, we describe every machine—its hardware, software, and purpose.

- *Autopolicy Server (ap-server)*. A server we created to provide the ap-switch with the management data, i.e., the profile that the switch should implement. The server is an Intel NUC machine running Ubuntu 18.04 LTS.
- *Autopolicy Switch (ap-switch)*. An Intel NUC running Ubuntu, used for easy setup and maintenance (in real setup the switch can be much less powerful). We used Open vSwitch v. 2.9.2 to implement an SDN switch, and emulated physical ports using dedicated VLANs for each device. For communication with the ap-server, we used a separate network plane.
- *Service Provider*: An Intel NUC running Ubuntu, on which we started a basic TCP server.
- *IoT Endpoint*. 3 Raspberry Pi 3B+ machines running Raspberry Pi OS, with an HTTP server for the Autopolicy identity.

### 6.3. Experimental Validation

For validating Autopolicy, we launched simple volumetric DDoS attacks from the IoT Endpoints against the Service Provider, using the testbed described in the previous paragraph (see Figure 6 and Figure 7). We conducted 2 experiments: one with Autopolicy disabled, and another one with Autopolicy enabled. The experiment scenario was as follows:

1 Period of time with stable network conditions.
2 DDoS attack made by the IoT Endpoints.
3 DDoS attack stops.
4 Period of time with stable network conditions.

To simulate the DDoS attack, as if some of the IoT Endpoint devices were part of a botnet, we used a traffic generator tool, scapy (see https://scapy.net). The tool was creating as many SYN packets as possible, in order to flood the Service Provider. Since we assumed the attacker did not know the exact port the Service Provider was listening on, the destination port on each packet was randomized.

During all the steps listed above, an ordinary user was repeatedly accessing the Service Provider—i.e., another IoT Endpoint, not in the botnet, was trying to use the service as usual. We measured the average service response time from this perspective, and plotted it versus time to analyze the effect of the DDoS attack.

**Figure 8:**
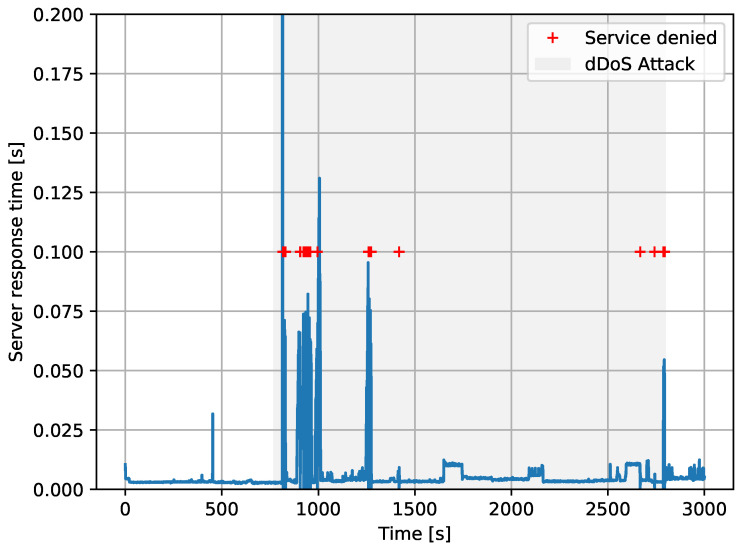
Autopolicy disabled. Grey area shows when the attack took place. Blue line shows the response time as seen from the user to the Service Provider. Red crosses are denied (timeout) services.

In Figure 8, we present the results of the first experiment, without Autopolicy. As can be seen, the service response time was much worse during the DDoS attack (grey background). Moreover, we saw the service was completely denied many times due to socket timeouts, so the ordinary user sometimes could not use the service during the attack at all.

For the second experiment with Autopolicy enabled, we implemented a traffic profile for the IoT Endpoints that lowered the maximum network throughput, and clipped the set of available TCP ports. We highlight that the same policy was enforced on all IoT Endpoints, regardless of whether they were part of the botnet or not, which includes the ordinary service user.

**Figure 9:**
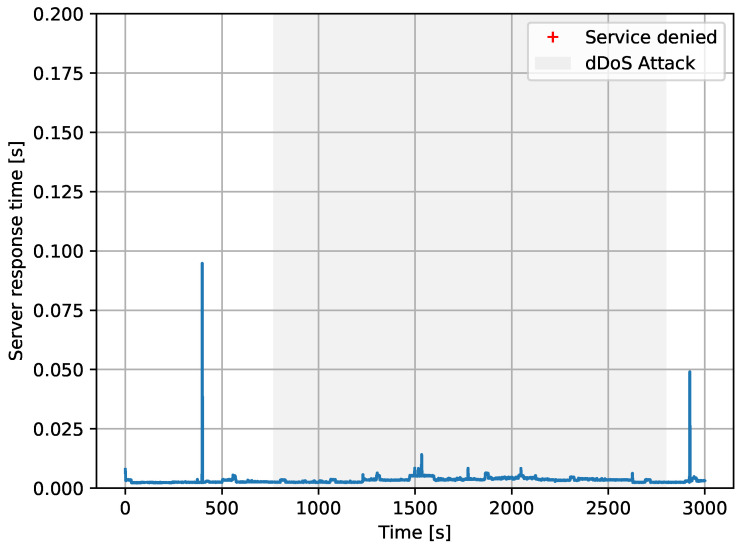
Autopolicy enabled. Grey area shows when the attack took place. Blue line shows the response time as seen from the user to the Service Provider.

Figure 9 presents the results. Contrary to the previous experiment, we did not see a considerable increase in the service response time. The user did not experience any connection timeouts, and the service remained completely responsive, which demonstrates the efficacy of Autopolicy. Note that we obtained this result while using the standard Linux traffic control mechanisms, so the additional overhead and energy consumption on the switch are similar to a regular, stateless Linux firewall.

## 7. Discussion

In this work, we gave a detailed description and prototype implementation of the fundamental functional blocks that are presented in Figure 1. We highlight this is the first paper on Autopolicy, which introduces the overall idea to the academic and research community. Future work is planned to extend upon our concepts, for instance we want to specify the Profile Builder using select traffic classification methods [37]. We also see potential to publish the traffic profiles using Peer-to-Peer (P2P) networks backed by the blockchain technology for data integrity, similar to the Google Certificate Transparency project or the Bitcoin cryptocurrency [38,39,40]. Both the architecture and prototype implementation support future extensions to our proposal in this paper.

Will IoT manufacturers adopt Autopolicy or a similar mechanism? We hope some will, especially given the existing standardization efforts at the IETF [21], but we argue the future networks for IoT sensors should be prepared for the opposite. We advocate against the default policy of no network usage limits for IoT devices, which should be replaced with an automated mechanism that will constrain their upstream IP traffic to reasonable limits. Because the history has already shown that IoT devices are widely open to hijacking via massive exploitation of security vulnerabilities [7], we claim that the only effective strategy to protect computer networks from IoT-launched attacks is to completely distrust the software running on IoT devices for that purpose. This is why, in an abstract sense, Autopolicy lets manufacturers run computer code on the network switches (SDN forwarders) that connect to the IoT devices that they produce. That is, it allows for pushing a crucial part of their network security function to the operator’s infrastructure—or, if the manufacturer does not cooperate—it allows for systematic and automated strategy for replacing the default "no limits" policy in a sensor network.

What is essentially novel in our paper? The concepts of traffic policing, firewalls, and Authentication, Authorization, and Accounting (AAA) [41] are already well-known in the literature. However, we believe its the new context of IoT security and enormous DDoS attacks [42] that came to old ideas. Thus, our contribution is a vision for integrated, systematic, and universal technique for defeating IoT-launched DDoS attacks. We also tried to keep Autopolicy as simple as possible—using only the popular network technologies—in order to alleviate its adoption and experimentation.

How does our work compare with MUD [21] (RFC8520)? As already mentioned in Section 2, Autopolicy was developed independently for edge security in a larger SerIoT project [5], and as such we consider our proposal complementary, not contrary. We believe it can foster the IETF standardization process and in general encourage better network operation practices. The unique feature of Autopolicy is that it explicitly discusses and addresses the need to limit the upload bandwidth of IoT devices hosted in an SDN network. In future we plan to integrate Autopolicy with the existing MUD data model. However, note that we consider additional network traffic aspects when compared with the MUD standard. These aspects are crucial for identifying potential network attacks or threats in an IoT network. Moreover, note that MUD makes use of device-emitted URLs only, whereas Autopolicy introduces a more general concept of device identities (which include the manufacturer URL). This allows for, e.g., better visibility into the network, more control over its traffic policing process, and the use of hierachical organization of traffic profiles (see Section 5.1). Finally, note that similarly to MUD, Autopolicy can talk to IoT devices using LLDP and other protocols (HTTP server on the device is not required, see Section 4.3).

How does Autopolicy integrate with the SDN concept? It was designed as an independent security model that works inside SDN networks without interfering with their mechanisms. Although in principle Autopolicy can be useful without the use of SDN, it can support various security mechanisms delivered by SDN networks—foremost, by the SerIoT project [43]. As future work, we plan on implementing the following ideas:

1 Applying OpenFlow rules on the edge forwarders (instead of the standard Linux tc tool), to configure traffic control mechanisms in SDN nodes.
2 Automatic creation of traffic profiles using traffic statistics collected by the SDN switch, e.g., IPFIX supported by Open vSwitch [22].
3 Notifying about violations of traffic profiles, for their analysis by additional SDN controller logic or operator action. In such a case, it is possible to include this information in the routing mechanism, influencing the selection of the packet route [5].

## 8. Conclusions

We introduced our proposal, called Autopolicy, to the academic and research community. The goal of Autopolicy is to provide network operators a means for automated and systematic deployment of traffic policing in an SDN-enabled sensor network. For that purpose, Autopolicy relies on manufacturers publishing IP traffic profiles for the IoT devices they produce, which allow limiting the available bandwidth and other network resources. This is primarily motivated by the fact that IoT devices often do not need to make use of the full potential of their network interfaces, while—at the same time—they often become hijacked and exploited for launching DDoS attacks and spreading malware on the Internet. Autopolicy also supports the situation in which IoT manufacturer does not publish a traffic profile for their devices. In such a case, the network operator can easily put default IP traffic restrictions on all devices, and use an automated traffic profile builder.

In this paper, we make use of SDN for the enforcement of IP traffic profiles, and we additionally evaluate our approach on a real scenario, which integrates the main Autopolicy components. As a future work, we plan to consider the use of blockchain to share traffic profiles among different manufacturers, as well as the use of machine learning techniques to identify advanced network attacks based on our proposed traffic profiles.
